# Effects of Habitat-Forming Species Richness, Evenness, Identity, and Abundance on Benthic Intertidal Community Establishment and Productivity

**DOI:** 10.1371/journal.pone.0109261

**Published:** 2014-10-14

**Authors:** Julie Lemieux, Mathieu Cusson

**Affiliations:** Département des sciences fondamentales, Université du Québec à Chicoutimi, Chicoutimi, Québec, Canada; University of Waikato (National Institute of Water and Atmospheric Research), New Zealand

## Abstract

In a context of reduced global biodiversity, the potential impacts from the loss of habitat-forming species (HFS) on ecosystem structure and functioning must be established. These species are often the main community primary producers and have a major role in the establishment of organisms through facilitation processes. This study focuses on macroalgae and mussels as HFS within an intertidal zone along the St. Lawrence estuary (Quebec, Canada). Over a 16-week period, we manipulated the *in situ* diversity profile (richness, evenness, identity, and abundance) of the dominant HFS (*Fucus distichus edentatus, F. vesiculosus*, and *Mytilus* spp.) in order to define their role in both the establishment of associated species and community primary production. Contrary to expectation, no general change in HFS richness, evenness, abundance, or identity on associated species community establishment was observed. However, over the study period, the HFS diversity profile modified the structure within the trophic guilds, which may potentially affect further community functions. Also, our results showed that the low abundance of HFS had a negative impact on the primary productivity of the community. Our results suggest that HFS diversity profiles have a limited short-term role in our study habitat and may indicate that biological forcing in these intertidal communities is less important than environmental conditions. As such, there was an opportunistic establishment of species that ensured rapid colonization regardless of the absence, or the diversity profile, of facilitators such as HFS.

## Introduction

Anthropogenic activities and climate change are the main drivers of global biodiversity loss *via* habitat destruction and modification [1]–[3]. These stressors negatively affect biodiversity-ecosystem functioning (BEF) relationships by altering the interaction between species [e.g. complementarity, 4] and decreasing habitat quality [5], thereby reducing ecosystem services, such as fisheries and enhanced coastal production and water purification provided by biodiversity [6]. Therefore, understanding the role of biodiversity in ecosystem functioning has become one of the main areas of focus in ecology [7]–[9].

Even though numerous studies have found a positive impact of diversity on ecosystem functioning [10]–[12], research results are not always consistent [e.g. 13]. A possible cause could be related to how the identity and the dominance structure, or evenness [14], may alter how the richness affects processes and functions within a species-rich community relative to a species-poor community [13]. It has been argued that changes in the dominance structure (evenness) may arise prior to biodiversity loss with consequences on ecosystem functions [15], including facilitation, which is a key mechanism positively influencing ecosystem efficiency through enhanced diversity [16]–[18]. Facilitation is provided by, among others, habitat-forming species [or ecosystem engineers sensu 19] that create or modify habitat. Habitat-forming species (hereafter HFS) have positive impacts on species richness and abundance, play a major role in organizing community structure, and have an important function in determining community productivity [6], [20].

In the marine intertidal zone, seaweeds [7], [21] and mussels [22] fulfill the role of HFS by increasing the complexity of these habitats [23]. Macroalgae offer protection against physical stresses, such as waves and air exposure [24]–[26]. They also provide a suitable environment for organisms by offering shelter and protection from predation [17] and desiccation [27], as well as serving as a food source [28], [29]. Mussel beds enhance diversity by creating a more heterogeneous substrate providing additional refuges for species to colonise [30]. Mussels also reduce wave swept impacts (hydrodynamic facilitation) allowing other species to colonize the bedrock [31].

As each HFS has a specific range of functional traits and a particular assemblage, [32], [33] a community with a higher HFS abundance and richness should increase the diversity of associated species. Also, increasing evenness should enhance the representation of each HFS [34] as well as the richness effect [35]. Increasing these characteristics should influence the establishment of associated species and their diversity [36]–[38]. Therefore, habitats marked by a high abundance, richness, and evenness (equal abundance) of HFS should support a more diverse assemblage of associated species.

Primary productivity of the whole community, as an ecosystem function, could also be influenced by changes in richness [39], evenness [40], identity [41], and abundance of HFS [42]. Primary production depends on the interaction of habitat complexity, shade, and nutrient enrichment [43], as well as algal diversity [8], [34], [40], [44]. These characteristics will act through complementarity [45] and the sampling effect. The complementarity effect occurs when a greater range of functional traits in a system allows a better use of resources [18], [46] by inducing better exploitation of niches and resources, thus making the whole community more efficient. Sampling effect is the natural selection of a more competitive or productive species. Increasing the richness enhances the probability of having one species that is more productive than the other species [47].

Diversity indice is composed of both richness and evenness components [14], [48], [49]. Disentangling their separate effects in studies of biodiversity-ecosystems functioning would be valuable. Richness and evenness have different roles in community functioning [50], [51] and should be treated separately [52]–[54]. For instance, species richness is responsible for the number of functional traits [55], while evenness may influence the richness effect by controlling the variation of traits represented in a community [35], [56]. In a rich community with high evenness, the chances of having a more productive species that is well represented will be greater than in a dominant community [57], [58]. Moreover, evenness is known to have a positive impact on productivity by increasing the representation of each species' functional traits [59] allowing a greater complementary effect.

In this paper, we designed an *in situ* experiment to test the effects of richness, evenness, abundance, and identity of three HFS on the establishment and characteristics of the associated species and the overall community primary productivity. We used realistic changes in HFS structure in a subarctic intertidal community where pronounced climate change impacts are expected, with increasing averages and variances of water and air temperatures; changes in salinity; and a thinner ice cover during a shorter winter period [60]. It is anticipated that high latitude habitats will experience stronger modifications in richness, composition, and abundance of HFS [61]–[63]. We hypothesised that increasing richness, evenness, and abundance of HFS within a community will stimulate the establishment of a more diverse community of associated species by enhancing habitat complexity, facilitation processes, and productivity through better niche partitioning and complementarity. We predicted that the identity of the HFS would affect the structure of their associated assemblage and the community function (e.g. productivity) due to their own physical and biological characteristics. A better understanding of how HFS diversity profiles affect communities will allow scientists to make better predictions and give more comprehensive recommendations to policy makers.

## Methods

### Site description

The study was located in the intertidal zone near the municipality of Sainte-Flavie (48°37′42.5″ N, 68°11′55.7″ W) along a straight coast on the south shore of the St. Lawrence estuary (Province of Quebec, Canada). No field permit was required in our study location, and no threatened or endangered species were involved. The coastal substrate is composed of stable bedrock moderately exposed to waves and with limited exposure to freshwater inflow and human disturbances. The water salinity ranges from 24 to 28 PSU and the average water level is 1.17 m above the lowest spring tide level with an average amplitude of 2.5 m. The annual water temperature varies between 4°C and 15°C (St. Lawrence Global Observatory; SLGO.ca). The shore communities can be exposed to moderate or heavy ice scouring [64], [65]. The experimental site was located in the mid-low intertidal zone where the fucoids (*Fucus distichus edentatus* and *Fucus vesiculosus*) are the dominant species of canopy macroalgae and the benthic flora and fauna are typical of a subarctic community [66].

### Experimental setup

To test the effect of habitat-forming species evenness, richness, identity, and abundance on associated species, artificial communities were assembled *in situ* in the intertidal zone (tidal height between 0.8–0.9 m). A total of 56 polyethylene experimental grids were screwed to the bedrock with a flat surface (30×30 cm; square mesh of 3.2 cm), and all organisms were removed by scraping. A minimum distance of 3 m between all grids was respected and they were all located in the mid-low intertidal zone where the maximum biomass is found. Habitat-forming species (HFS) from the same intertidal level were collected to assemble artificial communities. Mature individual plants (approximate size: 15 to 25 cm) of *Fucus distichus edentatus* and *Fucus vesiculosus* were harvested nearby. These two macroalgae might be considered redundant having the same functional role. We used these specific algae due to their high abundance on the shore, and they are representative of a subarctic environment. For the blue mussels (composed of *Mytilus edulis*, *M. trossulus*, and hybrids, hereafter named *Mytilus* spp.), individuals (shell length of 2.5 to 3.5 cm) were collected from a single mussel bed about 20 km away at the same tidal height and from similar environmental conditions to our experimental site. This was done for logistical reasons as the higher abundance of similar size mussels in the adjacent site was easier to harvest and allowed us to transplant them within 12 hours. All visible epibionts on macroalgae and mussels were removed gently by hand and attached individually to the grid using plastic coated wire according to each treatment (see details below for each treatment). A total of 70±5 g of mussels (about 20 individuals) was placed in 10×8 cm plastic mesh bags; each bag represented about 5% cover of the total 30×30 cm grid surface. To facilitate mussel byssal attachment and further collection of associated organisms, a rubber substrate was placed under the mussels in each bag. The bags were fixed to the grids using tie-wraps in a way to ensure that all mussel apertures were facing up.

The percentage cover of each HFS was manipulated in each grid to form different artificial assemblages resembling those observed in the surrounding communities. Six polyspecific treatments containing the three habitat-forming species were divided into two levels of total abundance; High (AH-) and Low (AL-) with ∼120% and ∼50% total cover respectively (sum of the % cover of all manipulated species on grid), and three levels of evenness among manipulated species; High (-JH), Medium (-JM), and Low (-JL) with obtained Pielou *J*' index values (average ± SD) of 0.98±0.01, 0.79±0.02 and 0.56±0.03 respectively (see Table 1). A *J*' index close to 1 means a more equal abundance among habitat-forming species, while a low value indicates dominance. Three monospecific treatments were also used with 100% cover for both *Fucus* species (FUVE, FUED) and 30% cover for the mussels (MYTI). The abundance values used in all abovementioned treatments for 3 HFS species are common for the surrounding area (e.g. individual species cover of 20–100% for both *Fucus* sp and 10–30% for mussels; Lemieux and Cusson unpublished data). *Fucus distichus edentatus* is often the dominant macroalgae at the tidal level of our experimental plots. Procedural controls with empty shells (treatment name: SHEL) in bags were used to separate the effect of the living mussels from their shells. Control plots with grids alone (without any HFS, treatment name: CONT) were also used. Finally, natural references (with at least >80% of *Fucus* spp., treatment name: NATU) were randomly sampled with a 30×30 cm quadrat on the same intertidal level. All treatments were randomly assigned to each grid. Six replicates were used for all polyspecific treatments and the natural reference treatments, while four replicates were used for the monospecific treatments, procedural empty shells control, and empty control grid treatments for a total of 62 experimental plots (i.e. 56 grids and 6 natural references).

**Table 1:** Composition of all treatments including the six artificial polyspecific and three monospecific communities for the three manipulated habitat-forming species: *Fucus distichus edentatus*, *Fucus vesiculosus* and *Mytilus* spp.

Note	Treatment name	Abundance (A)	Evenness (J)	*Fucus distichus edentates* (% cover)	*Fucus vesiculosus* (% cover)	*Mytilus* spp. (% cover)
Plurispecific	AHJH	High	High	50	50	30
Plurispecific	AHJM	High	Medium	80	30	15
Plurispecific	AHJL	High	Low	85	15	5
Plurispecific	ALJH	Low	High	20	15	15
Plurispecific	ALJM	Low	Medium	30	5	10
Plurispecific	ALJL	Low	Low	40	5	5
Monospecific	FUED			100	0	0
Monospecific	FUVE			0	100	0
Monospecific	MYTI			0	0	30
Empty mussel shells only	SHEL			0	0	30
Only grid	CONT			0	0	0
Natural community*	NATU					

*percentage covers of the three habitat-forming species were not manipulated.

The percentage covers that were used to create the three levels of evenness and two levels of abundance including information on the procedural controls (empty shells and grid) are shown. See methods section for details.

The experiment began on May 14^th^ 2011 and remained in place until September 4^th^ 2011. Maintenance was done every two weeks to ensure that each treatment remained constant throughout the experiment. In early September, at the collecting time, visual evaluations of the percentage cover of each observed macroscopic (>1 mm) species were recorded using a 30×30 cm quadrat divided into 25 squares with values of 4% cover each. The total cover can easily exceed 100% since all organisms are counted (total abundance  =  sum of all species % cover). Thereafter, in all experimental plots, each macroalgae, mussel bag, grid, and organism attached to the rock was collected separately, in individual bags, and brought to the laboratory. Loosely attached organisms (and associate sessile organisms on them) caught by the grids were considered separately (see results section below). In the laboratory, the HFS were gently washed with filtered saltwater over a 0.5 mm mesh sieve. All associated biota were preserved in 70% ethanol for further sorting. All organisms were identified to the lowest possible taxa level (usually species), counted, and weighed (maximum precision: 0.0005 g). Additional identified species in the laboratory were added to the field visual evaluation data. For those species, that were usually very small, we used a transformation into percentage cover by multiplying the number of individuals per species by an arbitrary value of 0.01%. All biomass values were converted into energy (kJ) using published mass-to-energy conversion coefficients [67]. No biomass was measured for the encrusting species (e.g. *Ralfsia* spp. or barnacles) due to their nature. A biomass (kJ) data set was used together with the % cover data set for further precision in community abundance structure in further univariate (e.g. evenness and diversity indices) and in multivariate analyses. Animals were classified based on their trophic guilds: Grazers: 8 species; Filter feeders: 5 species; Omnivores: 11 species (see Table S1). These three groups were chosen in order to have a maximum density of species within them.

### Production measurement

During the maximum growth period in mid-July 2011, the primary production of the whole community was measured in all treatments (three randomly chosen replicates) by monitoring the change in CO_2_ mole fraction (ppm) *in situ* using a benthic chamber [method and devices described in 68]. The benthic chamber is made of a transparent Plexiglas box, with a 30×30 cm base, covered with a dome; the chamber's total volume is 18 L, and it is connected through a closed circuit to a CO_2_ infrared gas analyser (LI-COR Inc, LI-820, Lincoln, NE, USA). The data were recorded on a data logger (LI-COR LI-1400; LI-COR Inc.) every 15 seconds (mean of 5 sec data interval) during a 10 to 20 minute incubation depending on the community response. Measurements were carried out with ambient daylight (always over 1000 µmol photon/m^2^) to measure the net primary productivity (NPP) and in the dark (benthic chamber covered with an opaque polyethylene sheet) to measure the respiration (R). The gross primary production (GPP) was calculated by adding NPP to R. This method was not used to evaluate the total budget of the shore community, but it gives an accurate and useful measure of primary production at the community scale in similar conditions.

### Data analyses

All analyses were done on the community of associated species, which excluded the three manipulated habitat-forming species, except for new recruits. The data analyses were done using a two-step approach. First, a two-way ANOVA was done on the polyspecific treatments only to analyse the main fixed factors of abundance and evenness treatments and their interaction. This allowed testing for the abundance factor regardless of evenness levels and vice versa. Since none of the results were significant in the first two-way ANOVA approach, one-way ANOVA (and, consequently, one-way PERMANOVA for multivariate analyses, see below) comparing all treatments were done (fixed factor, 12 treatment levels) on total abundance (sum of species % cover or biomass in kJ), richness, Pielou evenness (*J*'), and Shannon-Wiener diversity (*H*'; Log_e_) for each plot. ANOVA assumptions were checked by a graphical examination of the residuals [69], followed by multiple comparison tests (Tukey-HDS, unless stated) when necessary. One-way ANOVA was performed on the NPP, R, and GPP values among treatments (nine levels see results section).

The structure (using raw data) and the composition (with presence/absence) of communities and trophic guilds were compared among treatments using Bray-Curtis similarity into Permutational multivariate analysis of variance [PERMANOVA; 70]. In some cases, when only a restricted number of permutations were possible, Monte Carlo p-values (named *p_mc_*) were used [71]. Principal coordinate ordinations (PCO) were used to visualize the multivariate data (results of the PCO in Fig. S1 and S2). The contribution of each species to the average Bray-Curtis dissimilarity among treatments was assessed (SIMPER analyses, PRIMER). Further analyses were done on the abundance among trophic guilds (e.g. Grazers, Filter feeders, and Omnivores) using the PERMANOVA pairwise test. Univariate analyses were done using JMP 10.0 (SAS Institute, Cary, NC), while multivariate analyses and ordinations were done in PRIMER + PERMANOVA 6.1 (Plymouth Marine Laboratory, UK). A significance level α = 0.05 was used for all statistical tests.

## Results

On the collection date in September 2011, we observed a total of 45 associated species (algae: 13; animals: 32) with an average (± SE) of 14±3 by experimental plot (habitat-forming species excluded). An average total abundance cover of 94±38% (algae: 58%; animals: 37%) for associated species was observed in each experimental plot (see Table S2). Note that total abundance can be over 100% since it represent the sum of all species % cover.

**Figure 1:**
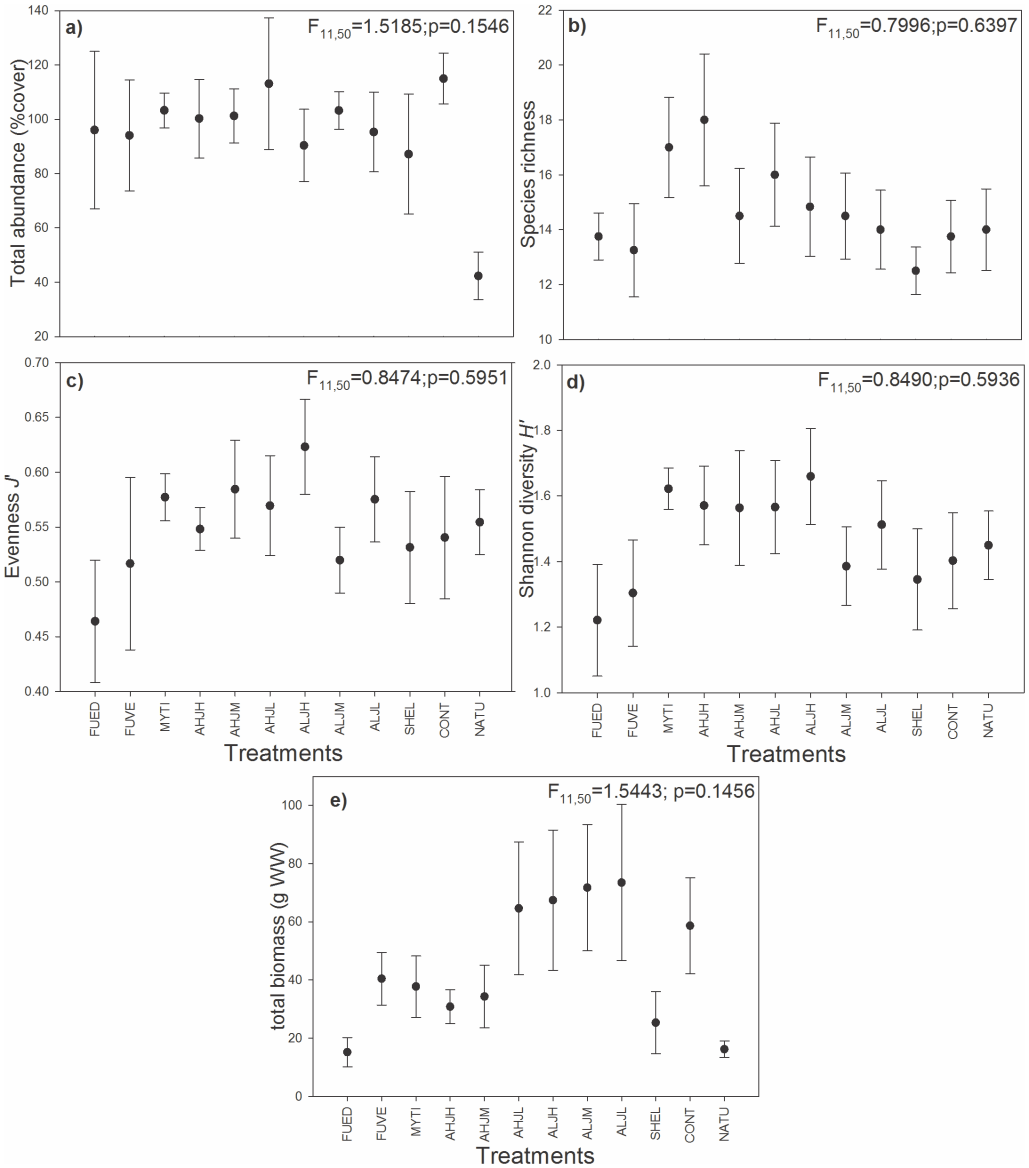
Average values (± SE) of a) total abundance (% cover), b) species richness, c) evenness (Pielou *J*'), and d) diversity index (Shannon *H*') e) in total abundance in biomass (g of Wet Weight) of associated species for each treatment. Treatments consisted of artificial assemblages with habitat-forming species having 2 levels of abundance (high, AH: 100–130 total % cover; and low, AL: 40–45% cover) and three levels of evenness *J*' values (high ±0.097: JH; medium ±0.75: JM; and low ±0.55: JL) as well as monoculture treatments with 100% cover of *Fucus distichus edentatus* (FUED), 100% cover of *Fucus vesiculosus* (FUVE), 30% *Mytilus* spp. (mussel), and a control with 30% *Mytilus* spp. empty shells (shells) and a natural reference community (natural). Percentage cover data set was used here, see Methods section.

No differences in total abundance, species richness, evenness, or Shannon diversity of the associated species were observed among all treatments (Fig. 1). Also, varying dominance structure and richness in habitat-forming species (HFS), and their identity in the monospecific treatment, did not change the abundance structure (pseudo-*F*_10,45_ = 1.20; *p* = 0.2110) or the composition (data transformed in presence/absence; pseudo-*F*_10,45_ = 0.76; *p* = 0.8570) of associated species. Moreover, our results show that the colonization in CONT and SHEL treatments did not produce differences in the community properties compared with the presence of any HFS in mono- or polyspecific treatments (Fig. 1). When each of the HFS monospecific treatments was contrasted separately, the Shannon diversity of associated species MYTI is higher than FUED treatments (*t*-ratio  = 3.42; *p* = 0.0110). Similar results obtained with biomass (kJ) were analysed (detailed results and figures not shown).

The encrusting algae *Ralfsia clavata* covered up to 80% of the rock surface under the experimental grids in all treatments. This alga rarely covered more than 10% in the natural community. Indeed, when we contrasted *R. clavata* cover between NATU and all other treatments, its percentage cover was marginally different (*p*<0.05). By removing *R. clavata* from the analyses, the structure within the assemblage in the AHJH treatment became different from the two macroalgal monospecific treatments, FUED (*t*-ratio  = 2.20, *p* = 0.0210) and FUVE (*t*-ratio  = 1.88, *p* = 0.0170), and became marginally different from the ALJL treatment (*t*-ratio  = 1.61, *p* = 0.0840) and MYTI treatment (*t*-ratio  = 1.72, *p* = 0.0590) (see Fig. S1). *Gammarus* spp. and recruits of *Fucus* spp. are the main taxa responsible for the difference between these treatments, respectively explaining up to 33% and 22% of differences. FUED and FUVE have more *Fucus* recruits than AHJH, while the latter has more *Gammarus* spp. and *Mytilus* spp. Analyses of the composition did not show significant results (see Fig. S1).

The separate collection of the organisms that were loosely attached to or caught by the grid (including various sessile organisms that were not attached to habitat-forming species or the ground; e.g. organisms within or on detritus or macrophyte species that were not present in the experimental site tide level) allowed us to remove them from the data set and perform again the same analyses. Without this “grids effect” and *Ralfsia* spp, the structure of associated species in AHJH remained different from FUED and FUVE. The treatments SHEL, CONT. and MYTI showed a difference in structure with AHJH (t = 2.04, *p* = 0.0420; t = 1.81, *p* = 0.0490 and t = 1.81, *p* = 0.0330 respectively). Moreover, AHJH showed a marginally different assemblage structure from ALJH and ALJL (t = 1.55, *p* = 0.0740 and t = 1.57, *p* = 0.0690 respectively; Fig. S2). The variability between these treatments is explained by many species, but the two main species responsible for the differences were the gastropods *Lacuna vincta* and *Margarites helicinus*, respectively explaining up to 6% and 4% of differences. The same pattern emerges when analysing the data in kJ.

### Difference in trophic guild

**Figure 2:**
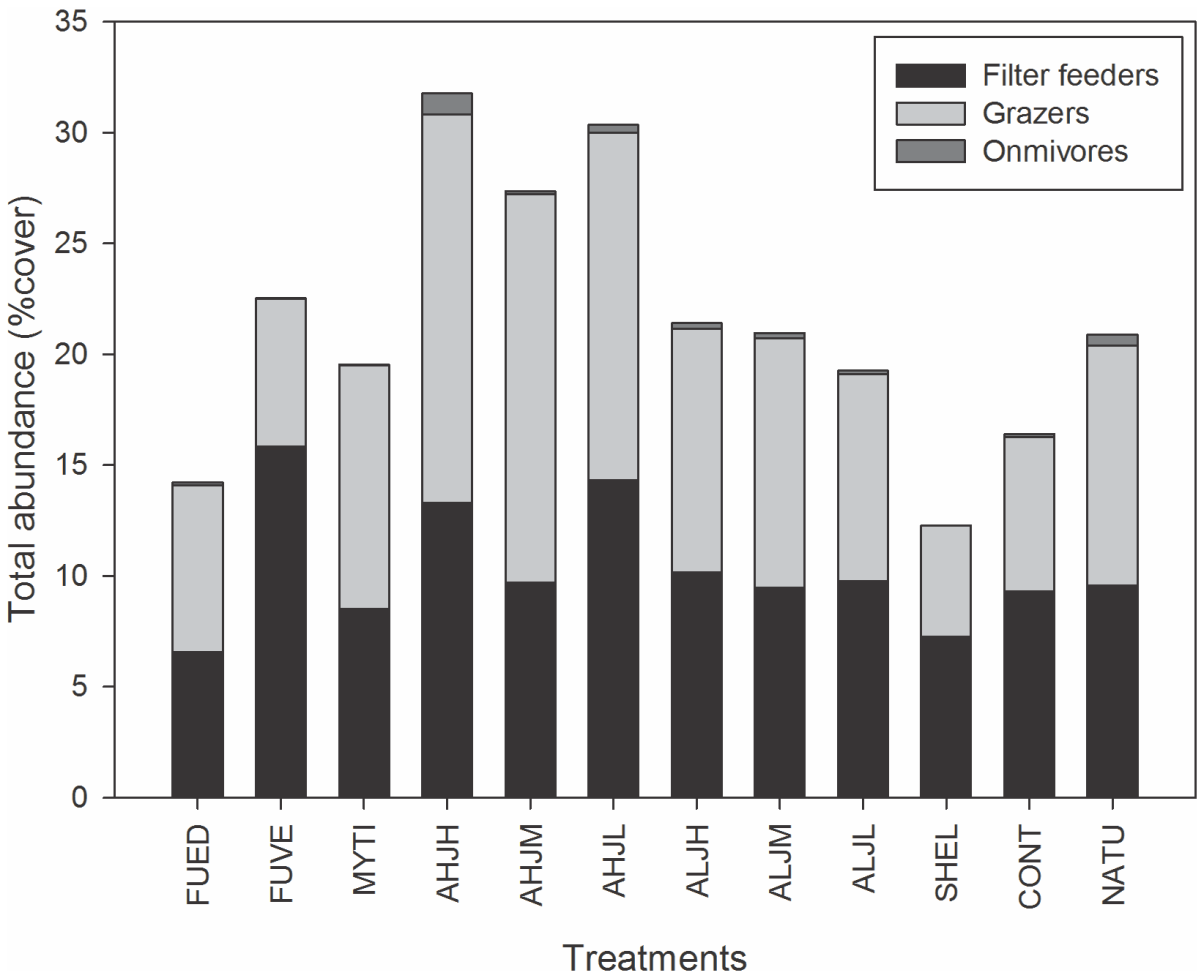
Distribution of total abundance in percentage cover of three trophic guilds among treatments: Grazers (8 species), Filter feeder (5 species) and Omnivores (11 species). See Table 1 for the details of the treatments and Table S1 for details of the trophic guilds group composition.

We first compared the three trophic guilds together among the treatments and no difference in their structure was found (Pseudo-*F*_11,50_ = 1.064; *p* = 0.389; Fig. 2). Second, we analyzed each trophic guild separately and compared them among treatments. We did not observe an effect of richness, evenness, or identity of the HFS on the Grazers (total abundance: *F*_11,50_ = 0.92; *p* = 0.5257 and richness: *F*_11,50_ = 1.08; *p* = 0.3978), Filter feeders (total abundance: *F*_11,50_ = 1.12; *p* = 0.3665 and richness: *F*_11,50_ = 1.51; *p* = 0.1571), and Omnivores (total abundance: *F*_11,50_ = 1.33; *p* = 0.2341 and richness: *F*_11,50_ = 0.85; *p* = 0.5925).

Our results show no significant difference among treatments for the Filter feeders in terms of structure or composition. There was, however, a difference in structure of the Grazers between FUVE, FUED, and SHEL treatments (*t* = 2.25, *p_mc_* = 0.0400; *t* = 2.90, *p_mc_* = 0.0190). The omnivores showed differences in structure between some treatments. FUED is different from AHJH (*t* = 2.60; *p_mc_* = 0.0060), AHJM (*t* = 3.23; *p_mc_* = 0.0050), and MYTI (*t* = 2.51; *p_mc_* = 0.0230), and marginally different from FUVE (*t* = 1.97; *p_mc_* = 0.0600). FUVE is different from AHJH (*t* = 2.52; *p_mc_* = 0.0070), AHJM (*t* = 2.67; *p_mc_ = *0.0190), and ALJM (*t* = 1.96; *p_mc_* = 0.0470).

### Primary production

**Figure 3:**
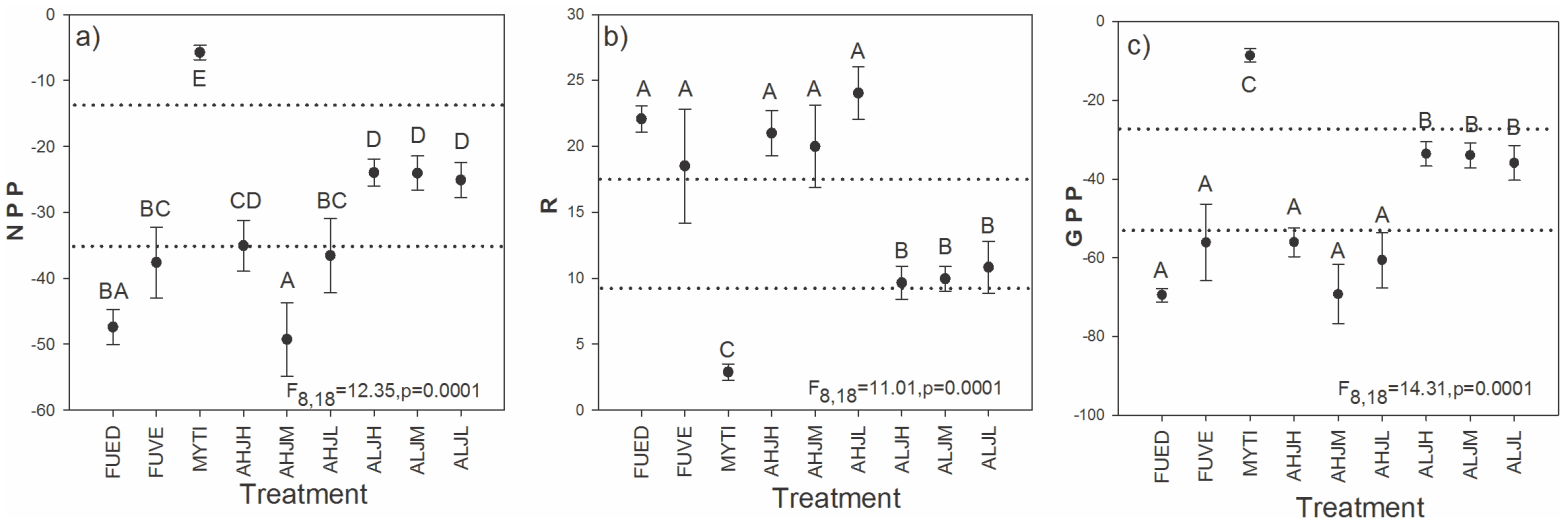
Average (± SE) values of productivity variables (µmolCO_2_*mlO_2_^−1^*min^−1^) of a) Net primary production (NPP), b) community respiration (R) and the c) gross primary production (GPP). Measurements were taken in July 2011 from each treatment and from 3 randomly chosen replicates (see Methods). The dotted lines represent the confidence interval (±95%) of the production done on natural community (see results section for details). Levels not connected by the same letter are significantly different. See Table 1 for the details of the treatment.

All productivity variables (Net primary production: NPP; community Respiration: R; and Gross Primary Production: GPP  =  NPP+R) included plots with the three manipulated HFS. At the time of the measurement in July 2011, we observed an average (± SE) of 8±2 species by experimental plot and an average % cover of 101±41 (without HFS: richness  = 6±2; abundance  = 21±20. The average richness for algae and animals was 4±1. The Spearman correlation (0.63) between the community in July (time of the production measurement) and September (end of the experiment) showed a high similarity, meaning that the community at the time of measurement and at the end of the experiment remained mostly the same. In figure 3, the dotted lines represent the primary production of natural communities (± CI95% of values obtained during July 2010 for 20 natural plots on the same site). For the same area, the natural level of productivity is a little higher than our experimental plots. We consider that this measure gives good estimates of the primary productivity of natural communities during the summer 2011.

The three variables of the primary production measurement were all compared among treatments. MYTI (mussels alone) has the lowest NPP, R, and GPP of all treatments (Fig. 3). The R and GPP showed large differences between the two levels of abundance tested regardless of evenness levels. The two macroalgae have the same R and GPP values level than the high abundance treatments. However, for the NPP some differences occur within the high abundance treatments. The monospecific treatment of FUVE and FUED showed similar NPP. FUED were different compared with all other treatments (except AHJM). FUVE is the same as all other high abundance treatments (except AHJM).

## Discussion

In this paper, we attempted to define the role of richness, evenness, identity, and abundance of habitat-forming species (HFS) on their associated species and community productivity. Generally, our results do not support our hypotheses that predicted a positive effect of the HFS diversity profile (richness, evenness, abundance, identity) on the characteristics of associated species. However, we did observe an effect of HFS richness on the structure of the associated species. Interestingly, all monoculture treatments showed differences in the structure of their grazer and omnivorous guilds but not in filter feeders. The effect of HFS on community functions was solely driven by the abundance of HFS macroalgae that increased community productivity variables.

### Effect of HFS on richness, evenness, identity, and abundance of associated species

In our experiment we did not observe a broad impact of HFS richness, evenness, identity, and abundance on associated species characteristics. We are confident that these non-significant results were not due to the sample size used (n = 4 and 6, see Methods), as *post hoc* power analyses indicated that, depending on a variable considered, a sample size varying between 12 and 305 would have been required to get significant results (detailed analyses not shown). Redundancy between the two *Fucus* species, inherent in the experimental design, would suggest that as long as one species can compensate for the loss or decline of the other, there will be no difference in community processes as theoretically predicted [72]. However, mussel and macroalgae treatments (FUED, FUVE, and MYTI) resulted in the same associated species characteristics after 16 weeks of colonization. This was contrary to our expectations because mussels change the heterogeneity of the rocky bottom surface by retaining sand and allowing species like Polychaeta to settle into the mussel bed [73]. A fully structured soft-bottom community naturally associated with mussel beds may not have had time to become established during one season, thus explaining why we did not see different assemblages between the macroalgae (FUED and FUVE) and MYTI treatments. Also, the treatment with empty *Mytilus* spp. shells (SHEL) presented a community with characteristics similar to one with living mussels (MYTI). Indeed, by increasing the heterogeneity of the substrate, empty shells do provide refuge for organisms from predation, wave shock, and desiccation, which makes the shell substrate just as important as living mussels [74], [75].

Our experiment was designed to test potential effects in the mid-low intertidal zone where the macroalgae canopy biomass was maximal, and offers constant optimized protection for understory organisms. It is at this intertidal zone that we usually observe a high diversity of associated fauna (personal observations). The link between HFS diversity and their associated species might have depended upon the tidal level considered [27]. Indeed, in the low intertidal zone, biotic factors control the community, while in the high intertidal zone abiotic factors control the community [26], [76]. In the high intertidal zone, harsher environmental conditions prevail and the protective influence of habitat-forming species is greater [77].

### Effect of HFS on the structure and composition of associated species

Although we did not observe much effect of the HFS diversity profile on the aggregated characteristics of the associated community, the effects on abundance structure (multivariate) were, however, detected. This was somewhat expected as when assemblages are compared, univariate tests (species independent) are often less sensitive than multivariate ones [species dependent; 78]. In our study, evenness in HFS abundances influenced the structure of the associated species. Indeed, in the AHJH treatment, where the three HFS were present in almost equal proportions, the structure of the associated species was different compared with the three HFS in the monospecific treatments (FUED, FUVE, MYTI). The three main species responsible for the differences in structure between the monospecific and the polyspecific treatments were *Gammarus* spp., *Mytilus* spp., and *Fucus* recruits. FUED and FUVE have more *Fucus* recruits than AHJH, while the latter has more *Gammarus* spp. and *Mytilus* spp. recruits. The *Gammarus* spp. would prefer the complex environment offered by the polyspecific AHJH treatments since they feed on small invertebrates, worms, small algae, and detritus [79], which are probably in greater abundance amongst mussels and protected by the macroalgae against predation and desiccation at low tide [80]. On the other hand, the absence of the whiplash effect [sensu 28] from macroalgal fronds in the MYTI treatment would enhance the establishment of new individuals of *Fucus* spp., as seen in our results.

The observed effects on the structure were interesting as they suggest a link with the increased complexity induced by the mussel bed and macroalgae canopy cover present in the AHJH treatment. This difference was not found with our low abundance assemblages and lower evenness (ALJL) among the three HFS. Our results are in accordance with other studies which found an impact of a change in the identity (macroalgae species with morphological difference) of seaweed on structure but not on community characteristics (univariate) of richness and abundance of invertebrate epifauna [36].

### Analysis by trophic guilds

The diversity profile of HFS did not have any effect on the abundance structure in guilds and within each guild separately among all treatments. Nonetheless, the HSF diversity profile affected the abundance structure of both grazers and omnivores, while it did not affect the filter feeders. Since each species offers different functional traits, richness triggers a greater range of functional traits [81]. In our study, changing the structure within a functional group could possibly affect the functions in the community in a longer term. The incorporation of functional group analyses (as with trophic guilds) in BEF studies increase the chance of identifying potentially key mechanisms that would otherwise be missed with only the analysis of the components of diversity [82], [83]. Our results suggest that if the HFS diversity profiles were modified, as in our treatments, the ability of each functional group (or trophic guild) to carry out their functions (e.g. grazing activity, decomposition, etc.) within the community would be affected. This would be worth to be addressed in a longer term experiment.

### Effect of HFS on primary productivity

We did not observe an effect of richness or evenness on the productivity values (net primary production: NPP; respiration: R; and gross primary production: GPP) of communities, whereas, theoretically, the values should, increase with producer species richness [84], [85]. The productivity variables were positively influenced by the total abundance of the two manipulated macroalgae. Indeed, the most abundant treatments that included macroalgae (monospecific FUVE and FUED and polyspecific AH-) had higher values of R and GPP than all of the low abundance treatments (polyspecifics AL-). For the AHJM and AHJL treatments, NPP values were higher than all low abundance treatments except for AHJH. The AHJH treatment had the same NPP as the low abundance treatments; in part, this may be due to the inclusion of a greater proportion of FUVE, which is more associated with lower NPP values (although not statistically significant) than FUED. In this regard, similar responses in productivity between our HFS algae, *Fucus distichus edentatus* and *Fucus vesiculosus*, would additionally support their status as a redundant species in our system. Nevertheless, our results strongly suggest that their high abundance levels in nature are critical for the whole shore productivity.

### Perspectives on biodiversity relationships in the subarctic context

In our study area, ice-scouring episodes in the spring can partially reset the benthic community [86]. The succession pattern following such an event implies that the community species richness, abundance, and identity changes throughout the summer [87]. It is possible that the general richness and evenness effect of habitat-forming species (HFS) become more important in a well-established and less disturbed community. This may explain, in part, why our 16-week experiment may not have detected all potential HFS diversity effects.

The link between the diversity of HFS and their associated species is based on species relationships and interactions. The chances of having stronger interactions among species generally increases with diversity [88]. Therefore, the removal of important species can lead to indirect effects with a cascading loss of species through a series of secondary extinctions [28], [89], [90]. These changes in the interactions among species will first influence the structure of the community before an actual species loss or exclusion takes place. This might be the reason why we detected effects on abundance (multivariate) structures but not on richness or total abundance. In their review, Hillebrand et al. [15] predicted that a change in dominance would occur before loss of species with consequences in abundance structure (dominance/evenness), species interactions, and community processes within the ecosystems. Our results showed that these structural changes within abundant species would not have much effect on short-term species establishment. Further investigations at larger scales (site and regional scale) are needed to better predict large changes within assemblages. However, manipulative studies are difficult or impossible at larger scales. Indeed, most manipulative studies have been done at a limited spatial scale [e.g. meter–scale 21] and temporal consequences of the diversity effect may either be seen only after a few years, but also the effect may be greater or null thereafter [91]. Our observed HFS richness effect on some abundance structures of the newly established community may just be an indication that the effects of the HFS diversity profile generate complex responses within the associated community. Consequently, longer experiments would have helped to understand further diversity interactions. But, in the subarctic environment studied, this was not possible due to macroalgal senescence, very harsh autumn conditions, and ice cover in winter. Also, small differences in proportional densities of HSF among our polyspecific treatments (see Table 1) may have slightly affected our analyses. Additional tests with varying assemblages within levels of abundances/evenness could be evaluated in future to gain insight to this potential effect. Further extension of such *in situ* manipulative studies to higher intertidal levels where environmental conditions are more hasher (as previously discussed above), and other marine habitats, would certainly add to our understanding of the role of habitat-forming species in maintaining local biodiversity levels. Nevertheless, the linkage of biodiversity with ecosystem function must also be understood in environmentally driven habitats. The strength of the compensatory dynamics that influence community stability varies with latitude. Compensatory dynamics within assemblages can also be influenced by the HFS as they control the associated species [92]. Moreover, the canopy removal effect on community stability is a function of latitude and environmental forcing [92], [93].

Our study demonstrate the need for *in situ* experiments that reflect real-life interactions among species is crucial in order to better assess the role of biodiversity on ecosystem functioning and the potential effect of species abundance structures changes on their community functions.

## Concluding Remarks

In this work, the effects of richness, evenness, identity, and abundance of habitat-forming species (HFS) on the diversity and establishment of associated species were studied over a 16-week period in a subarctic environment. There was an effect of the HFS richness and evenness on the abundance structure of the associated species but not on their aggregative community characteristics (richness, total abundance, diversity, etc.). These results support the idea that local loss of a HFS would first promote changes in the abundance structure before changes in the composition community, including species extinction. Moreover, the study of the richness effect alone in biodiversity/ecosystem functioning studies would only focus on one important, but incomplete, component of biodiversity. Richness effect studies, when coupled with other aspects of diversity such as evenness, allow the exploration of the effect of different mechanisms on community processes. To our knowledge, our *in situ* study in a subarctic environment is one of the first to examine the effects of richness, evenness, identity and abundance of habitat-forming species on associated community structure and productivity. This research represents a step forward to a better understanding of the general effect of biodiversity on community dynamics.

## Supporting Information

Figure S1 **Percentage cover data set: PCO (Bray Curtis similarity) showing the difference in a) structure and b) composition and without** ***Ralfsia Clavata*** **in c) structure and d) composition of the associated species among treatments.** See figure 1 in article for details on the treatments.
(TIF)

Figure S2 **Percentage cover data set without the grid effect: PCO (Bray Curtis similarity) showing differences in a) structure and b) composition.** The encrusting species *Ralfsia Clavata* is not included in the analysis. See figure 1 in article for details on the treatments.
(TIF)

Table S1 **Taxa list of all observed organisms at the end of the experiment (September 2011).** For the animals, trophic guilds in which they were classified are shown.
(DOCX)

Table S2 **Average (± SE) of percentage cover for each taxa in all treatments.**
(DOCX)
