# Supplementary material for: Effects of Habitat-Forming Species Richness, Evenness, Identity, and Abundance on Benthic Intertidal Community Establishment and Productivity
Source: PLoS One. 2014 Oct 14;9(10):e109261. doi: 10.1371/journal.pone.0109261 (PMC4196772; doi:10.1371/journal.pone.0109261)
Supplement: Table S1 — Taxa list of all observed organisms at the end of the experiment (September 2011). For the animals, trophic guilds in which they were classified are shown. (DOCX) [file pone.0109261.s003.docx]

**Table S1.** Taxa list of all observed organisms at the end of the experiment (September 2011). For the animals, trophic guilds in which they were classified are shown.

| **Algae** | **Species name** | **Phylum** | **Class** | **Order** | **Family** | **Genus** | **Species** |
| --- | --- | --- | --- | --- | --- | --- | --- |
| **Brown algae** | *Fucus distichus edentatus* | Phaeophyta | Phaeophyceae | Fucales | Fucaceae | *Fucus distichus* | *edentatus* |
|  | *Fucus vesiculosus* | Phaeophyta | Phaeophyceae | Fucales | Fucaceae | *Fucus* | *vesiculosus* |
|  | *Ascophyllum nodosum* | Phaeophyta | Phaeophyceae | Fucales | Fucaceae | *Ascophyllum* | *nodosum* |
|  | *Chordaria flagelliformis* | Phaeophyta | Phaeophyceae | Ectocarpales | Chordariaceae | *Chordia* | *flagelliformis* |
|  | *Ectocarpus spp.* | Phaeophyta | Phaeophyceae | Ectocarpales | Ectocarpaceae | *Ectocarpus* |  |
|  | *Ralfsia fungiformis* | Phaeophyta | Phaeophyceae | Ectocarpales | Ralfsiaceae | *Ralfsia* | *fungiformis* |
|  | *Ralfsia clavata* | Phaeophyta | Phaeophyceae | Ectocarpales | Ralfsiaceae | *Ralfsia* | *clavata* |
|  | *Scytosiphon lomentaria* | Phaeophyta | Phaeophyceae | Scytosiphonales | Scytosiphonaceae | *Scytosiphon* | *lomentaria* |
|  | *Petalonia fascia* | Phaeophyta | Phaeophyceae | Scytosiphonales | Scytosiphonaceae | *Petalonia* | *fascia* |
| **Red algae** | *Hildenbrandia prototypus* | Rhodophyta | Rhodophyceae | Cryptonemiales | Hildenbrandiaceae | *Hildenbrandia* | *prototypus* |
|  | *Porphyra spp.* | Rhodophyta | Rhodophyceae | Bangiales | Bangiaceae | *Porphyra* |  |
|  | *Clathromorphum circumscriptum* | Rhodophyta | Rhodophyceae | Corallinales | Corallinaceae | *Clathromorphum* | *circumscriptum* |
|  | *Rhodomela confervoides* | Rhodophyta | Rhodophycaea | Ceramiales | Rhodomelaceae | *Rhodomela* | *confervoides* |
|  | *Polysiphonia spp.* | Rhodophyta | Rhodophyceae | Ceramiales | Rhodomelaceae | *Polysiphonia* |  |
| **Green algae** | *Ulvaceae* | Chlorophyta | Chlorophyceae | Ulotrichales | Ulvaceae |  |  |
|  | *Ulothrix sp.* | Chlorophyta | Chlorophyceae | Ulotrichales | Ulotrichaceae | *Ulothrix* |  |
| **Trophic guild** | |  |  |  |  |  |  |
| **Filter feeders** | *Aulactinia stella* | Cnidaria | Anthozoa | Actiniaria | Actiniidae | Aulactinia | stella |
|  | *Macoma Baltica* | Mollusca | Bivalvia | Veneroida | Tellinidae | Macoma | baltica |
|  | *Mytillus edulis* | Mollusca | Bivalvia | Filibranchia | Mytilidées | Mytillus | edulis |
|  | *Mya arenaria* | Mollusca | Bivalvia | Myoida | Myidae | Mya | arenaria |
|  | *Skeneopsis planorbis* | Mollusca | Gastropoda | Neogastropoda | Skeneosidae | Skeneopsis | planorbis |
|  | *Pectinaria gouldii* | Annelida | Polychaeta | Canalipalpata | Pectinariidae | Pectinaria | gouldii |
|  | *Balanus crenatus* | Arthropoda | Maxillopoda | Sessilia | Balanidae | Balanus | crenatus |
|  | *Semibalanus balanoides* | Arthropoda | Maxillopoda | Sessilia | Archaeobalanidae | Semibalanus | balanoides |
| **Grazers** | *Littorina obtusata* | Mollusca | Gastropoda | Neotaenioglossa | Littorinidae | Littorina | obtusata |
|  | *Littorina saxatilis* | Mollusca | Gastropoda | Neotaenioglossa | Littorinidae | Littorina | saxatilis |
|  | *Littorina littorea* | Mollusca | Gastropoda | Neotaenioglossa | Littorinidae | Littorina | littorea |
|  | *Lacuna vincta* | Mollusca | Gastropoda | Neotaenioglossa | Littorinidae | Lacuna | vincta |
|  | *Margarites helicinus* | Mollusca | Gastropoda | Archaeogastropoda | Trochidae | Margarites | helicinus |
|  | *Tectura testudinalis* | Mollusca | Gastropoda | Patellogastropoda | Lottidae | Tectura | testudinalis |
|  | *Jaera marina* | Arthropoda | Malacostraca | Isopoda | Janiridae | Jaera | marina |
| **Omnivores** | *Nucella lapillus* | Mollusca | Gastropoda | Neogastropoda | Muricidae | Nucella | lapillus |
|  | Sipuncula | Sipuncula | Sipunculidea |  |  |  |  |
|  | Oligochaeta | Annelida | Clitellata |  |  |  |  |
|  | Polychaeta | Annelida | Polychaeta |  |  |  |  |
|  | *Nereis* spp. | Annelida | Polychaeta | Aciculata | Nereididae | Nereis |  |
|  | *Eteone longa* | Annelida | Polychaeta | Aciculata | Phyllodocidae | Eteone | longa |
|  | *Fabricia sabella* | Annelida | Polychaeta | Canalipalpata | Sabellidae | Fabricia | sabella |
|  | *Sabellaria* | Annelida | Polychaeta | Canalipalpata | Sabellidae |  |  |
|  | *Polydora* spp. | Annelida | Polychaeta | Canalipalpata | spionidae | Polydora |  |
|  | *Polynoidae* spp. | Annelida | Polychaeta | Aciculata | Polynoidae |  |  |
|  | *Lepidonotu squamatus* | Annelida | Polychaeta | Aciculata | Polynoidae | Lepidonotus | squamatus |
|  | *Phyllodocidae spp.* | Annelida | Polychaeta | Aciculata | Phyllodocidae |  |  |
|  | *Capitellidae* | Annelida | Polychaeta |  |  |  |  |
|  | *Gammarus* spp. | Arthropoda | Malacostraca | Amphipoda |  |  |  |
|  | *Cancer irroratus* | Arthopodes | Malacostraca | Decapoda | Cancridae | Cancer | irroratus |
