# Supplementary material for: Effects of Habitat-Forming Species Richness, Evenness, Identity, and Abundance on Benthic Intertidal Community Establishment and Productivity
Source: PLoS One. 2014 Oct 14;9(10):e109261. doi: 10.1371/journal.pone.0109261 (PMC4196772; doi:10.1371/journal.pone.0109261)
Supplement: Table S2 — Average (± SE) of percentage cover for each taxa in all treatments. (DOCX) [file pone.0109261.s004.docx]

**Table S2.** Average (±SE) of percentage cover for each taxa in all treatments.

| **Treatments** | **AHJH** | | **AHJM** | | **AHJL** | | **ALJH** | | **ALJM** | | **ALJL** | | **CONT** | | **FUED** | | **FUVE** | | **MYTI** | | **NATU** | | **SHEL** | |
| --- | --- | --- | --- | --- | --- | --- | --- | --- | --- | --- | --- | --- | --- | --- | --- | --- | --- | --- | --- | --- | --- | --- | --- | --- |
|  | **mean** | **±SE** | **mean** | **±SE** | **mean** | **±SE** | **mean** | **±SE** | **mean** | **±SE** | **mean** | **±SE** | **mean** | **±SE** | **mean** | **±SE** | **mean** | **±SE** | **mean** | **±SE** | **mean** | **±SE** | **mean** | **SE** |
| **Macroalgea** |  |  |  |  |  |  |  |  |  |  |  |  |  |  |  |  |  |  |  |  |  |  |  |  |
| *Fucus* spp | **3.10** | **4.00** | **6.45** | **7.00** | **21.27** | **16.67** | **14.95** | **16.17** | **8.75** | **8.83** | **22.03** | **21.83** | **18.10** | **29.75** | **14.45** | **18.75** | **8.69** | **9.25** | **9.54** | **13.75** |  |  | **4.24** | **15.00** |
| *Ascophyllum nodosum* |  |  |  |  |  |  | **1.22** | **0.50** |  |  |  |  | **5.00** | **2.50** |  |  |  |  |  |  |  |  |  |  |
| *Laminaria* spp |  |  |  |  | **0.41** | **0.17** | **2.04** | **0.83** |  |  |  |  |  |  |  |  |  |  |  |  |  |  | **0.50** | **0.25** |
| **Ephemeral algae** |  |  |  |  |  |  |  |  |  |  |  |  |  |  |  |  |  |  |  |  |  |  |  |  |
| *Enteromorpha* spp |  |  |  |  |  |  | **0.41** | **0.17** |  |  |  |  |  |  |  |  |  |  |  |  |  |  |  |  |
| *Antithamnion* spp | **0.05** | **0.03** | **0.82** | **0.33** |  |  |  |  |  |  |  |  |  |  |  |  |  |  |  |  |  |  |  |  |
| *Chordaria flagelliformis* | **1.55** | **1.00** | **1.22** | **0.50** | **3.21** | **2.50** | **3.60** | **1.83** | **0.82** | **0.33** | **1.67** | **1.00** | **2.87** | **1.75** | **15.50** | **7.75** | **0.75** | **0.38** | **3.00** | **1.50** |  |  | **2.31** | **2.00** |
| *Porphyra* spp | **0.84** | **0.50** | **2.81** | **1.50** | **1.33** | **1.17** | **6.69** | **4.00** | **1.03** | **0.67** | **8.76** | **6.67** | **1.00** | **0.50** | **0.48** | **0.38** |  |  | **4.50** | **3.75** | **4.08** | **1.67** | **0.50** | **0.25** |
| *Rhodomela confervoides* | **1.17** | **0.75** |  |  |  |  | **1.20** | **0.58** | **1.60** | **0.75** |  |  | **0.25** | **0.13** |  |  |  |  |  |  |  |  |  |  |
| *Ulvaceae* | **2.58** | **2.33** | **3.49** | **2.17** | **2.11** | **1.42** | **1.97** | **1.50** | **0.42** | **0.25** |  |  | **1.80** | **1.38** |  |  |  |  | **1.44** | **0.88** |  |  | **0.95** | **0.63** |
| **Encrusting algae** |  |  |  |  |  |  |  |  |  |  |  |  |  |  |  |  |  |  |  |  |  |  |  |  |
| *Clathromorphum circumscriptum* |  |  | **1.22** | **0.50** |  |  |  |  |  |  |  |  |  |  |  |  |  |  |  |  | **<0.00** | **<0.00** |  |  |
| *Hildenbrandia prototypus* |  |  |  |  |  |  | **<0.00** | **<0.00** |  |  |  |  |  |  |  |  |  |  |  |  | **<0.00** | **<0.00** |  |  |
| *Ralfsia clavata* | **25.70** | **34.42** | **6.59** | **44.33** | **28.34** | **43.00** | **10.34** | **28.17** | **10.41** | **50.67** | **23.14** | **35.33** | **28.10** | **51.50** | **42.08** | **59.25** | **37.17** | **53.75** | **5.48** | **47.00** | **10.81** | **9.00** | **31.61** | **46.25** |
| *Ralfsia fungiformis* |  |  |  |  |  |  |  |  |  |  |  |  |  |  |  |  |  |  |  |  | **0.20** | **0.08** |  |  |
| **Grazers** |  |  |  |  |  |  |  |  |  |  |  |  |  |  |  |  |  |  |  |  |  |  |  |  |
| *Gammarus* spp | **12.25** | **25.00** | **11.75** | **16.96** | **11.97** | **17.42** | **14.05** | **14.79** | **12.03** | **20.29** | **14.98** | **10.71** | **12.61** | **10.81** | **1.80** | **3.19** | **3.14** | **7.75** | **9.74** | **16.31** | **11.51** | **10.02** | **13.66** | **10.13** |
| *Jaera marina* | **0.24** | **0.58** | **0.25** | **0.40** | **0.21** | **0.26** | **0.17** | **0.39** | **0.20** | **0.33** | **0.21** | **0.26** | **0.11** | **0.11** | **0.02** | **0.03** | **0.11** | **0.10** | **0.39** | **0.55** | **0.22** | **0.35** | **0.10** | **0.11** |
| *Lacuna vincta* | **0.01** | **0.02** | **0.01** | **0.01** | **0.01** | **0.01** | **0.02** | **0.01** | **0.02** | **0.01** | **<0.00** | **<0.00** | **0.01** | **<0.00** | **0.01** | **0.01** | **0.04** | **0.03** | **0.01** | **<0.00** | **<0.00** | **<0.00** | **0.01** | **<0.00** |
| *Littorina littorea* | **0.01** | **<0.00** |  |  |  |  |  |  |  |  | **0.01** | **0.01** |  |  |  |  |  |  |  |  |  |  |  |  |
| *Littorina obtusata* | **6.18** | **8.27** | **2.78** | **5.67** | **5.76** | **9.21** | **3.38** | **5.21** | **3.41** | **5.01** | **4.57** | **5.48** | **4.03** | **4.88** | **2.33** | **3.79** | **5.43** | **11.58** | **0.57** | **3.27** | **2.98** | **6.04** | **3.18** | **3.56** |
| *Littorina saxatilis* | **2.28** | **4.36** | **1.03** | **3.46** | **2.19** | **4.80** | **2.46** | **4.52** | **2.12** | **4.03** | **2.87** | **3.98** | **3.17** | **4.31** | **1.61** | **2.75** | **1.57** | **4.06** | **0.83** | **4.45** | **1.15** | **3.11** | **2.89** | **3.48** |
| *Margarites helicinus* | **0.09** | **0.05** | **0.39** | **0.18** | **0.07** | **0.05** | **0.05** | **0.04** | **0.17** | **0.10** | **0.04** | **0.02** |  |  |  |  | **0.11** | **0.06** | **0.17** | **0.22** | **0.15** | **0.07** | **0.18** | **0.09** |
| *Tectura testudinalis* | **0.02** | **0.01** | **0.02** | **0.01** | **0.01** | **0.01** | **0.01** | **<0.00** | **<0.00** | **<0.00** | **0.01** | **0.01** | **0.01** | **0.01** |  |  | **0.01** | **<0.00** | **0.01** | **0.01** | **0.01** | **0.01** | **0.03** | **0.02** |
| **Filter feeders** |  |  |  |  |  |  |  |  |  |  |  |  |  |  |  |  |  |  |  |  |  |  |  |  |
| *Macoma Baltica* | **0.01** | **0.01** |  |  |  |  |  |  | **0.01** | **0.01** | **0.01** | **<0.00** |  |  | **0.01** | **0.01** | **0.02** | **0.01** | **0.02** | **0.01** |  |  |  |  |
| *Mya arenaria* |  |  | **0.02** | **0.01** |  |  |  |  |  |  |  |  |  |  |  |  |  |  |  |  | **<0.00** | **<0.00** |  |  |
| *Mytillus* spp | **11.25** | **15.18** | **9.31** | **13.24** | **12.08** | **12.07** | **8.53** | **10.48** | **8.77** | **10.80** | **4.52** | **6.73** | **3.61** | **3.39** | **5.86** | **5.22** | **2.19** | **5.66** | **4.52** | **10.24** | **9.19** | **10.82** | **3.05** | **2.64** |
| *Balanus* spp | **2.27** | **2.34** | **4.66** | **4.26** | **6.26** | **3.59** | **0.45** | **0.50** | **0.78** | **0.46** | **3.90** | **2.63** | **4.30** | **3.58** | **3.81** | **2.29** | **0.82** | **1.00** | **0.29** | **0.76** | **0.01** | **<0.00** | **3.78** | **2.38** |
| *Pectinaria gouldii* | **0.01** | **0.01** |  |  |  |  |  |  |  |  | **<0.00** | **<0.00** |  |  |  |  |  |  |  |  | **0.01** | **0.01** |  |  |
| *Skeneopsis planorbis* | **0.01** | **0.01** | **<0.00** | **<0.00** | **0.02** | **0.01** | **0.01** | **0.01** | **0.01** | **<0.00** | **0.01** | **<0.00** | **0.01** | **0.01** |  |  | **0.01** | **<0.00** | **0.01** | **<0.00** | **0.02** | **0.01** | **0.03** | **0.01** |
| **Omnivores** |  |  |  |  |  |  |  |  |  |  |  |  |  |  |  |  |  |  |  |  |  |  |  |  |
| *Oligochaeta* | **0.01** | **0.01** | **0.02** | **0.01** | **0.04** | **0.03** | **0.02** | **0.01** | **0.01** | **0.01** | **0.02** | **0.02** | **0.03** | **0.02** | **0.01** | **0.02** | **0.03** | **0.03** | **0.01** | **0.01** | **0.19** | **0.18** |  |  |
| *Polychaeta* |  |  |  |  |  |  |  |  | **<0.00** | **<0.00** |  |  |  |  | **0.01** | **<0.00** |  |  |  |  |  |  | **0.02** | **0.01** |
| *Acaria* | **0.02** | **0.01** | **0.07** | **0.03** | **0.10** | **0.07** | **0.02** | **0.01** | **0.01** | **0.01** | **<0.00** | **<0.00** |  |  | **0.03** | **0.02** | **0.06** | **0.04** | **0.01** | **0.01** | **<0.00** | **<0.00** |  |  |
| *Aulactinia stella* | **0.01** | **<0.00** | **<0.00** | **<0.00** |  |  |  |  |  |  |  |  |  |  |  |  |  |  |  |  | **<0.00** | **<0.00** |  |  |
| *Capitellidae* |  |  | **<0.00** | **<0.00** |  |  |  |  | **0.01** | **<0.00** |  |  |  |  |  |  |  |  | **0.02** | **0.01** |  |  |  |  |
| *Chironomidea larva* | **0.03** | **0.01** | **0.01** | **0.01** | **0.01** | **0.01** |  |  | **0.12** | **0.05** | **0.02** | **0.01** |  |  | **0.04** | **0.02** | **0.01** | **<0.00** | **0.01** | **<0.00** | **0.04** | **0.02** | **0.03** | **0.01** |
| *Eteone longa* |  |  |  |  |  |  |  |  |  |  | **0.01** | **<0.00** |  |  |  |  |  |  |  |  | **0.01** | **0.01** |  |  |
| *Fabricia sabella* | **0.01** | **0.01** |  |  | **0.01** | **<0.00** | **0.04** | **0.02** |  |  | **0.01** | **<0.00** |  |  |  |  | **0.03** | **0.02** | **0.01** | **0.01** | **0.10** | **0.09** |  |  |
| *Foraminifera* | **0.25** | **0.27** | **0.25** | **0.32** | **0.23** | **0.23** | **0.25** | **0.27** | **0.24** | **0.30** | **0.26** | **0.33** | **0.05** | **0.04** | **0.04** | **0.04** | **0.24** | **0.14** | **0.24** | **0.38** | **0.17** | **0.41** | **0.26** | **0.28** |
| *Lepidonotu squamatus* | **0.02** | **0.01** |  |  | **0.01** | **0.01** | **0.01** | **0.01** | **0.02** | **0.01** | **<0.00** | **<0.00** | **0.01** | **<0.00** |  |  |  |  | **0.01** | **0.01** | **0.04** | **0.02** |  |  |
| *Nereis* spp | **1.80** | **0.92** | **0.14** | **0.13** | **0.58** | **0.33** | **0.25** | **0.21** | **0.40** | **0.21** | **0.21** | **0.13** | **0.25** | **0.13** | **0.14** | **0.13** |  |  |  |  | **0.38** | **0.33** |  |  |
| *Phyllodocidae* spp |  |  |  |  |  |  |  |  |  |  |  |  |  |  |  |  |  |  |  |  | **<0.00** | **<0.00** |  |  |
| *Plathelminth* | **0.09** | **0.11** | **0.13** | **0.09** | **0.06** | **0.06** | **0.20** | **0.11** | **0.08** | **0.07** | **0.08** | **0.07** | **0.14** | **0.09** | **0.04** | **0.12** | **0.08** | **0.12** | **0.08** | **0.09** | **0.04** | **0.02** | **0.05** | **0.04** |
| *Polydora* spp | **0.01** | **<0.00** |  |  |  |  |  |  |  |  |  |  |  |  |  |  | **0.01** | **<0.00** |  |  | **<0.00** | **<0.00** |  |  |
| *Polynoidae* spp |  |  | **<0.00** | **<0.00** |  |  |  |  |  |  | **<0.00** | **<0.00** |  |  |  |  |  |  |  |  | **<0.00** | **<0.00** |  |  |
| *Sabellaria* spp |  |  |  |  |  |  |  |  |  |  |  |  |  |  |  |  |  |  |  |  | **<0.00** | **<0.00** |  |  |
| *Sipuncula* | **0.03** | **0.02** | **0.07** | **0.07** | **0.03** | **0.02** | **0.05** | **0.03** | **0.02** | **0.01** | **0.02** | **0.02** | **0.02** | **0.04** | **0.03** | **0.02** | **0.07** | **0.04** | **0.03** | **0.06** | **0.02** | **0.01** | **0.02** | **0.02** |
